# Adult Food Allergy Is an Under-Recognized Health Problem in Northwestern Mexico: A Population-Based Cross-Sectional Survey

**DOI:** 10.3390/epidemiologia6040085

**Published:** 2025-12-02

**Authors:** Lizbeth Vizcarra-Olguin, Marcela de Jesús Vergara-Jiménez, Juancarlos Manuel Velásquez-Rodríguez, Oscar Gerardo Figueroa-Salcido, Elisa María Barrón-Cabrera, Perla Y. Gutiérrez-Arzapalo, Fernando Salas-López, Noé Ontiveros, Jesús Gilberto Arámburo-Gálvez

**Affiliations:** 1 Nutrition Sciences Postgraduate Program, Faculty of Nutrition and Gastronomy Sciences, Autonomous University of Sinaloa, Culiacan 80019, Sinaloa, Mexico; lizvizolg@gmail.com (L.V.-O.); mjvergara@uas.edu.mx (M.d.J.V.-J.); oscar.figueroa@uas.edu.mx (O.G.F.-S.); elisabarron@uas.edu.mx (E.M.B.-C.); 2 Pediatric Hospital of the Sinaloa State, Culiacan 80200, Sinaloa, Mexico; dr.juancarlosvelasquez@gmail.com; 3 Integral Postgraduate Program in Biotechnology, Faculty of Chemical and Biological Sciences, Autonomous University of Sinaloa, Ciudad Universitaria, Culiacan 80010, Sinaloa, Mexico; 4 Center for Research and Teaching in Health Sciences, Autonomous University of Sinaloa, Culiacán 80030, Sinaloa, Mexico; perla.gutierrez@uas.edu.mx; 5 Department of Health Sciences, Universidad Autónoma de Occidente, Culiacán 80020, Sinaloa, Mexico; fernando.salas@uadeo.mx; 6 Department of Chemical, Biological, and Agricultural Sciences (DC-QB), University of Sonora, Navojoa 85880, Sonora, Mexico

**Keywords:** food allergy, adults, self-reported, anaphylaxis, prevalence, food allergen, survey

## Abstract

Objectives: To estimate the prevalence, clinical characteristics, and risk factors of IgE-mediated food allergy (FA) in adults from northwestern Mexico. Methods: A population-based, cross-sectional study was conducted in Culiacán, Sinaloa. A validated questionnaire was administered to 834 adults (valid response rate: 87.5%) in public spaces. Prevalence rates (95% CI) were calculated, and associations were analyzed using odds ratios (OR). Results: The prevalence rates of “current immediate-type FA”, “food-induced anaphylaxis” and adult-onset “current immediate-type FA” were 5.75% (4.27–7.49), 2.5% (1.75–4.10) and 2.99% (1.94–4.39), respectively. The most common allergens were shellfish (2.14% (1.28–3.39)) and milk (1.19% (0.57–2.19)). Epinephrine was prescribed in 9.5% of the cases with anaphylaxis history. General practitioners made the diagnosis of 63.4% of the FA cases. FA was associated with personal and family history of atopy. Conclusions: Adult FA is frequent in the population studied and could be an emerging public health problem, characterized by a high rate of adult-onset cases and suboptimal management of anaphylaxis.

## 1. Introduction

Food allergy (FA) is an adverse and reproducible immune-mediated reaction that occurs in sensitized individuals following ingestion, contact with, or inhalation of a food allergen [1]. The clinical manifestations of FA range from mild symptoms, such as urticaria, to severe reactions, including life-threatening anaphylaxis [2]. Emergency medications, such as epinephrine, are required to manage symptoms when accidental exposures occur [3]. The most common foods triggering allergy reactions are milk, wheat, egg, soy, peanuts, tree nuts, fish, and shellfish [4]. Avoidance of the offending food remains the only effective strategy for preventing allergic reactions. However, individuals with FA face a constant risk of unintentional exposure to food allergens through undeclared allergens in processed foods, cross-contamination, or inadequate cleaning of equipment [5,6]. These risks can significantly affect quality of life, causing anxiety, social limitations, and financial burden, especially among those with multiple food allergies or recurrent severe reactions [7,8].

FA affects more than 220 million people worldwide and it is believed that more children (8%) than adults (3–4%) suffer from FA [9]. Prevalence data have informed public health policies, such as the inclusion of sesame in mandatory food labeling regulations. Particularly, FA prevalence in adults has been well documented in Europe [10] and the United States [11], but data from developing countries remain scarce. In Mexico, population-based epidemiological studies on FA have primarily focused on pediatric [12,13,14] or adolescent populations [15]. Regarding adults, available studies remain limited, with a geographical focus on central and western Mexico and methodological diversity. For instance, research includes analyses of sIgE sensitization patterns in clinically referred cohorts of all ages [16], surveys using broad definitions of food hypersensitivity conducted via fixed quota sampling in recreational areas among adults aged 18–50 [17], comparisons of adverse reaction trends using similar public space sampling [18], and surveys focused specifically on young adults (18–25 years) in university settings, employing stricter symptom criteria [19]. To address the knowledge gap regarding IgE-mediated FA across the adult population (≥18 years) within the understudied northwestern Mexican region, the aim of the present study was to estimate the prevalence, clinical characteristics, and risk factors of immediate-type FA in the adult population of such a region by conducting a cross-sectional population-based survey.

## 2. Materials and Methods

### 2.1. Study Design and Population

A population-based, cross-sectional survey was conducted in Culiacán, Sinaloa, Mexico, between May and October 2024. Adults aged 18 years or older were invited to participate. A sample size of at least 710 participants was considered representative, assuming an anticipated prevalence of self-reported FA of 10.8% [11], a confidence interval of 99%, and a 3% margin of error.

Participants were approached in public locations (shopping malls, public parks, and squares) across different geographic areas of Culiacán, Sinaloa to ensure representation of diverse socioeconomic status (Figure S1). To capture a diverse population cross-section and minimize temporal bias, each location was visited on different days (at least one weekday and one weekend day) and during two distinct time shifts (9:00 AM–12:00 PM and 4:00 PM–6:00 PM). After the participants provided informed consent, trained personnel carried out face-to-face interviews using the SurveyMonkey platform. Incomplete questionnaires were excluded from the analysis.

### 2.2. Questionnaire

A structured and validated questionnaire, designed to estimate the prevalence of parent-reported FA, was adapted to ask questions directly to the interviewees (Supplementary Files S1 and S2). The unadapted version of the instrument has been utilized in Mexico and other Latin American countries [12,14,20,21]. Adaptations primarily involved adjusting wording for self-reporting while retaining the core structure for collecting demographic, clinical and family history information on atopic diseases. It also collected information about the type of signs/symptoms experienced, the timing of symptom onset, the number of reaction episodes, emergency care utilization, medical diagnoses, and treatment received in cases reporting adverse reactions to specific foods.

### 2.3. Case Definitions

Participants were classified according to previously described criteria [14]: (1) Adverse food reaction: Any self-reported recurrent adverse reaction triggered after ingestion of a specific food, regardless of the underlying mechanism. (2) Physician-diagnosed FA, ever: A positive response to the question “Has a doctor ever told you that you have food allergy?”; (3) Immediate-type FA, ever: Recurrent symptoms that are consistent with immediate-type hypersensitivity (e.g., skin rash, angioedema, respiratory symptoms, gastrointestinal symptoms, or cardiovascular involvement) and that occur within 2 h after food ingestion. This definition allows for the detection of up to 93% of subjects with relevant specific IgE to the implicated foods [22,23]; (4) Immediate-type FA, current: Participants meeting criteria for immediate-type FA, ever, and still reacting to the offending food. (5) Food-induced anaphylaxis: Defined as immediate-type FA with evidence of cardiovascular compromise or multisystem involvement, as per the World Allergy Organization criteria [24].

### 2.4. Statistical Analyses

Statistical analyses were performed using GraphPad Prism version 8.0 (GraphPad Software, San Diego, CA, USA). Descriptive statistics were used to summarize demographic and clinical variables. Prevalence rates were calculated using OpenEpi software version 3.03a and reported as percentages with 95% confidence intervals (CI). Associations between FA prevalence and clinical variables were assessed using Fisher’s exact test. The associations were presented as odds ratios (OR) with 95% CI calculated in a basic 2 × 2 table. A *p*-value < 0.05 was considered statistically significant.

### 2.5. Ethical Considerations

The study was conducted in accordance with the ethical principles of the Declaration of Helsinki. Participation was voluntary, and informed consent was obtained verbally. Before the interview, all participants received information regarding the study’s objectives, procedures, anonymity, and the confidentiality of all data, as well as that of the researchers and institutions involved. The Research Ethics Committee of the Autonomous University of Sinaloa approved the study protocol (approval number: 191-2025).

## 3. Results

### 3.1. Participants, Demographic and Clinical Characteristics

The valid response rate was 87.5% (834 valid questionnaires), and the proportion of women was 51.4% (n = 429). The mean age was 34 years (range: 18–93 years) and most participants had a college (45.9%) or a high school education (33.4%). The median completion time for the questionnaire was 3.8 min (interquartile range: 2.7–6.8 min) and the most frequently reported atopic conditions were allergic rhinitis (14.7%), drug allergy (14.0%), and atopic dermatitis (11.8%) (Table 1).

### 3.2. Prevalence Estimates

Among the 834 respondents, 120 reported adverse food reactions (14.4%, 95% CI: 12.1–17.0), of which 64 also reported symptoms consistent with an immediate-type FA (7.7%, 95% CI: 6.0–9.7). Of these 64, 16 informed that they had outgrown FA. Thus, the prevalence of “immediate-type FA, current” was 5.75% (n = 48; 95% CI: 4.3–7.5). Additionally, 41 participants (4.9%, 95% CI: 3.6–6.6) reported that a physician diagnosed them with FA. The prevalence of food-induced anaphylaxis was 2.5% (n = 21; 95% CI: 1.6–3.8) (Table 2). More women than men had a physician diagnosis of FA (7.2% (CI: 4.96–10.10) vs. 2.5% (CI: 1.19–4.49), *p* = 0.001) (Table 2).

### 3.3. Prevalence Estimates of Food Allergies for Specific Foods

**Figure 1:**
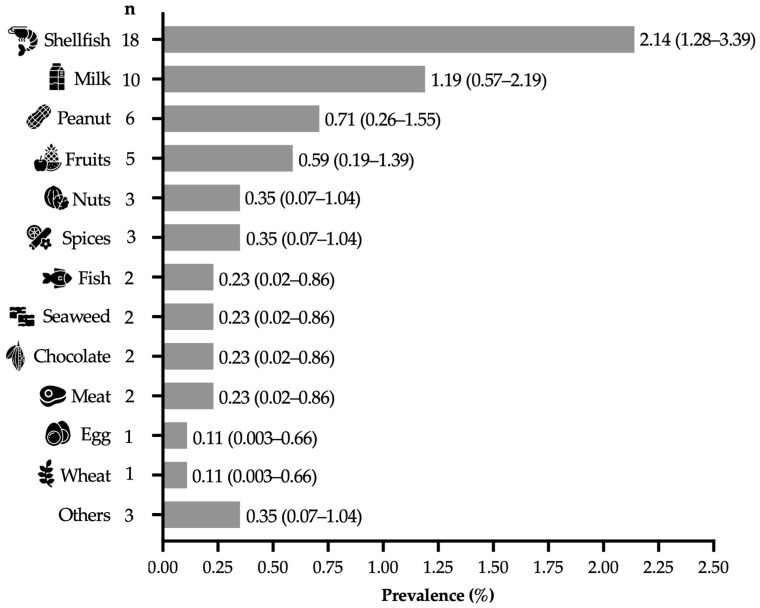
Food allergy prevalence by specific food in adults from northwestern Mexico (n = 834), presented as percentage and 95% confidence intervals.

Among the participants with “immediate-type FA, current” (n = 48), 16.6% (n = 8) reported reactions to two or more foods. The most common allergens were shellfish (2.15%, 95% CI: 1.28–3.39), milk (1.1%, 95% CI: 0.57–2.19), peanuts (0.71%, 95% CI: 0.26–1.55), and fruits (0.59%, 95% CI: 0.19–1.39) (Figure 1). Notably, 52.1% (n = 25) of the “immediate-type FA, current” cases reported the onset of their symptoms after the age of 18 (adult-onset FA prevalence of 2.99% (95% CI 1.9–4.3)). In these cases, the most common allergens were shellfish (48.0%), milk (20.0%), and peanuts (16.0%).

### 3.4. Clinical Characteristics and Context of Allergic Reactions

**Figure 2:**
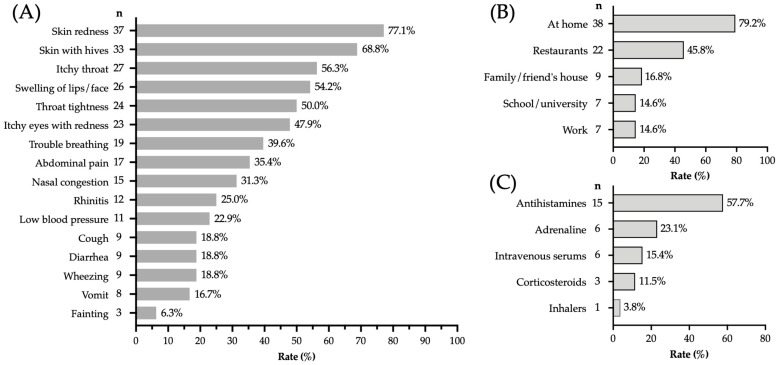
Clinical characteristics and context of allergic reactions in adults with “immediate-type FA, current”. (**A**) Frequency of signs and symptoms (n = 48). (**B**) Places where allergic reactions were triggered (n = 48). (**C**) Frequency of emergency treatments received when medical care was sought (n = 26).

The most frequent symptoms among the “immediate-type FA, current” cases (n = 48) were skin redness (77.1%) and urticaria (68.8%), followed by oropharyngeal symptoms like throat itching (56.3%), lip/face swelling (54.2%), and throat tightness (50.0%) (Figure 2A). Allergic reactions occurred mostly at home (79.2%) and in restaurants (45.8%) (Figure 2B). Fifty-four percent (n = 26) of the “immediate-type FA, current” cases required emergency medical care on at least one occasion, and antihistamines were reported as the main treatment received (57.5%) (Figure 2C). Among the 23 participants who met criteria for anaphylaxis, only 9.5% were prescribed injectable epinephrine.

### 3.5. Characteristics of Physician-Diagnosed FA Cases

General practitioners diagnosed most of the physician-diagnosed FA cases (63.4%), followed by allergists (31.7%). Clinical history was the most common diagnostic method reported (58.8%), followed by skin prick tests (26.8%), allergen-specific IgE (14.6%), and oral food challenge (9.8%).

### 3.6. Risk Factors Associated with Immediate-Type FA

A personal history of atopic diseases was associated with “immediate-type FA, ever”, including a history of non-food allergen-associated anaphylaxis (OR 5.96, 95% CI 2.59–13.50), animal allergy (OR 5.89, 95% CI 3.10–11.18), and allergic conjunctivitis (OR 4.14, 95% CI 2.20–7.81). Similarly, a paternal history of insect allergy (OR 8.24, 95% CI 1.43–40.78), maternal history of FA (OR 3.36, 95% CI 1.37–8.58) and a sibling history of allergic conjunctivitis (OR 4.26, 95% CI 1.65–11.2) were associated with the development of “immediate-type FA, ever” (Table 3).

## 4. Discussion

To our knowledge, the present population-based study provides the first estimate of FA prevalence in adults from northwestern Mexico. Certainly, population-based data on adult FA prevalence in Latin America are scarce, with most regional studies focusing on pediatric populations [25,26,27]. In the present study, the “immediate-type FA, current” prevalence was 5.8%. This adult prevalence rate is similar to the 5.9% prevalence of probable FA reported in adults from central Mexico [19], but higher than the 1.0% prevalence of probable IgE-mediated FA reported in adults from Central Brazil [28]. Others reported a prevalence rate of 14.9% for FA in an adult Colombian population; however, it is worth noting that the definition of FA used was based solely on self-reported adverse food reactions [29]. Outside the Latin American region, the adult FA prevalence rate estimated in the present study is lower than the rates reported in North Americans (10.8%) [11], similar to Canadians (5.7%), and falls within the upper end of the prevalence range for probable IgE-mediated FA (0.3% to 5.6%) reported in the EuroPrevall study [10]. Variations in prevalence rate estimations among survey studies are not uncommon and may be attributed to differences in dietary patterns and genetic predisposition of the studied population, as well as study designs and definitions.

Notably, the FA prevalence in the adult population from northwestern Mexico was higher than the prevalence rates reported in preschoolers (1.6%) [12] and schoolchildren (3.6%) [14] from the same geographical region and estimated using the same instrument. These data suggests that FA is not only persisting but also emerging in adulthood. Indeed, over half (52.1%) of the “immediate-type FA, current” cases reported in the present study began in adulthood, challenging the traditional view of FA as a predominantly pediatric condition [9], but in line with emerging international evidence [11,27]. Overall, the data highlight that adult-onset FA (prevalence 2.99%) is a common and under-recognized public health issue in adults from northwestern Mexico, whose mechanisms, potentially involving lifestyle factors, medication use, or changes in the gut microbiome that could alter immune tolerance in adults, require further investigation.

The most frequently reported food allergens were shellfish, milk, peanuts, and fruits. Particularly, the high prevalence of shellfish allergy (2.14%) was expected as the geographical area studied is considered a coastal region where the consumption of shellfish, either raw or lightly cooked, is high and shellfish allergy is common in the pediatric population [12,14]. Furthermore, the findings are consistent with those reported in pediatric populations from Latin American countries other than Mexico [21,30,31], but differ from those reported in many Western adult populations, where peanuts and tree nuts are among the most frequently reported food allergens [32,33]. Cultural issues, food availability, dietary patterns, environmental factors, and genetic load are aspects that can contribute to regional differences in prevalence estimations of specific food allergies, highlighting the need for local epidemiological supervision. Certainly, identifying common food allergens not covered by current mandatory labeling laws is essential for developing effective food safety policies for protecting the affected population.

The context where food allergic reactions were triggered reveals significant public health implications. The finding that 46% of the allergic reactions were triggered in restaurants is of particularly noteworthy, especially given the regulatory landscape. While Mexican federal laws [34] mandate the declaration of major allergens on prepackaged foods, there are currently no specific regulations for the food service sector in the country. Contrary, in other regions, such as the European Union, the legislation mandates clear allergen information for consumers in food services [35]. The absence of regulatory policies regarding food allergens information in food services places the burden of safety entirely on the consumer and creates a high-risk environment for individuals with FA, underscoring a clear need for policy development targeting the food service sector in Mexico. Mexican clinical guidelines [36] and other national consensus documents [37] recommend a detailed clinical history followed by diagnostic testing for FA diagnosis, as well as epinephrine as the first-line treatment for anaphylaxis. However, our study highlights a potential gap between these guidelines and clinical practice. The results show that general practitioners made most FA diagnoses (63.4%), relying primarily on clinical history rather than on the recommended confirmatory tests, suggesting possible misclassifications. Additionally, epinephrine was prescribed in only 9.5% of patients with a history of life-threatening anaphylaxis. These findings indicate critical deficiencies in both the diagnosis and emergency management of FA in this population. These findings underscore an urgent need for enhanced training for primary care physicians on accurate FA diagnosis and anaphylaxis management, alongside improved referral pathways to specialized allergy services. The low rate of epinephrine prescription is consistent with other studies conducted in the Latin American region [12,14,21,30,31]. Furthermore, a limited availability of epinephrine auto-injectors has been reported in Mexico [38], highlighting the need for public health policy actions to improve epinephrine access. Addressing these specific gaps in diagnostic procedures, access to specialists, and emergency readiness is essential for improving medical care for patients suspected of or living with food allergies.

The results also show that conditions such as anaphylaxis, animal allergy, and allergic conjunctivitis, as well as a maternal or sibling history of FA, significantly increase the odds for developing immediate-type FA. These findings are consistent with the concept of the “atopic march,” a well-documented progression where individuals with one allergic condition, such as atopic dermatitis, are predisposed to developing others, including FA and asthma [39]. Therefore, the results confirm the importance of obtaining a complete personal and family history of atopy during clinical evaluations to identify individuals at increased risk of developing FA who may benefit from earlier medical preventive counseling.

Finally, we should acknowledge that our study has some limitations. First, although a systematic sampling strategy was implemented to mitigate bias, including sites visited by people of diverse socioeconomic levels, which were visited more than once on different days (a weekday and a weekend) and at different times, probability or household sampling is recommended to improve the generalizability of the results. For example, homebound or elderly people, as well as rural populations, may be underrepresented. Second, self-report studies can lead to an overestimation of the true prevalence rates [40], and in the present study clinical confirmation of FA for all participants was not possible. Therefore, future studies on FA should consider evaluations with objective diagnostic criteria. And third, due to the study’s cross-sectional design, the identified associations cannot establish causality; the data were collected at a specific point in time, so the order of events cannot be established. However, our study has important strengths, including its population-based design in an understudied demographic group, the high response rate (reducing non-response bias), and the use of a validated questionnaire employing a strict case definition based on criteria highly predictive of IgE sensitization to minimize the overestimation of true FA prevalence.

## 5. Conclusions

Immediate-type FA in adults is common in northwest Mexico, affecting up to 5.75% of this population, with shellfish, milk, and peanuts being the most common allergenic foods. Furthermore, the study provides the first epidemiological evidence of adult-onset FA in Mexico (2.99%) and highlights that adherence to clinical guidelines for diagnosing and giving medical advice for managing food allergic reactions, especially in cases with a history of anaphylaxis, should be encouraged. Due to allergic reactions commonly occurring in restaurants, there is a need for regulatory policies for food services in Mexico to ensure the safety of diners living with FA.

## Figures and Tables

**Table 1:** Demographic characteristics of the participants.

Variable		n (%)
Average age in years (range)		34.4 (18–93)
Sex	Female	429 (51.43%)
	Male	405 (48.56%)
History of allergic diseases other than FA		
Allergic rhinitis		123 (14.74%)
Drug allergy		117 (14.02%)
Atopic dermatitis		98 (11.75%)
Chronic Urticaria		91(10.91%)
Conjunctivitis		68 (8.15%)
Asthma		59 (7.07%)
Animal allergy		53 (6.35%)
Insect sting allergy		39 (4.67%)
Anaphylaxis		23 (3.11%)

Acronymous: FA, Food allergy.

**Table 2:** Prevalence estimates stratified by sex.

Assessment	Number of Cases	Prevalence % (95% CI)			*p*
		Total (n = 834)	Men (n = 405)	Women (n = 429)	
Adverse food reactions	120	14.38 (12.08–16.96)	12.59 (9.52–16.22)	16.08 (12.73–19.91)	0.167
Physician-diagnosed FA, ever	41	4.91 (3.55–6.61)	2.46 (1.19–4.49)	7.22 (4.96–10.1)	0.001
Immediate-type FA, ever	64	7.67 (5.96–9.69)	6.17 (4.03–8.97)	9.09 (6.54–12.22)	0.12
Immediate-type FA, current	48	5.75 (4.27–7.49)	4.44 (2.65–6.93)	6.99 (4.76–9.83)	0.117
Food-induced anaphylaxis	23	2.75 (1.75–4.10)	1.72 (0.69–3.52)	3.72 (2.14–5.98)	0.081

Acronymous: FA, Food allergy; CI, Confidence Interval; PD, Physician-diagnosed; *p*, *p*-value for the comparison between men and women (Fisher’s exact test).

**Table 3:** Risk factors associated with immediate-type food allergy.

Allergic Disease	Immediate Type FA, Ever	No FA	*p*	Odds Ratio (CI 95%)
	(n = 64)	(n = 770)		
	n (%)	n (%)		
Personal history				
Anaphylaxis	8 (12.5)	18 (2.33)	0.0004	5.96 (2.59–13.50)
Animal allergy	15 (23.43)	38 (4.93)	<0.0001	5.89 (3.10–11.18)
Conjunctivitis	15 (23.43)	53 (6.88)	<0.0001	4.14 (2.20–7.81)
Allergic rhinitis	22 (34.37)	101 (13.11)	<0.0001	3.47 (1.95–5.93)
Urticaria	15 (18.75)	76 (9.87)	0.0026	2.79 (1.44–5.07)
Atopic dermatitis	15 (23.43)	83 (10.77)	0.007	2.53 (1.32–4.75)
Drug allergy	17 (26.56)	100 (12.98)	0.0075	2.42 (1.37–4.34)
Insect sting allergy	6 (9.37)	33 (4.28)	0.1115	2.31 (0.98–5.45)
Asthma	8 (12.5)	51 (6.62)	0.1205	2.01 (0.94–4.46)
Paternal history				
Insect sting allergy	2 (3.12)	3 (0.38)	0.049	8.24 (1.43–40.78)
Conjunctivitis	3 (4.68)	9 (1.16)	0.057	4.15 (1.18–15.67)
Asthma	5 (7.81)	21 (2.72)	0.042	3.023 (1.20–7.88)
Food allergy	6 (9.37)	29 (3.76)	0.044	2.64 (1.10–6.39)
Allergic rhinitis	4 (6.25)	24 (3.11)	0.263	2.07 (0.75–5.82)
Anaphylaxis	1 (1.56)	6 (0.77)	0.429	2.02 (0.17–12.58)
Drug allergy	4 (6.25)	33 (4.28)	0.519	1.48 (0.54–4.27)
Animal allergy	1 (1.56)	9 (1.16)	0.552	1.34 (0.12–8.35)
Atopic dermatitis	1 (1.56)	13 (1.68)	>0.99	0.92 (0.08–5.89)
Maternal history				
Food allergy	6 (9.37)	23 (2.98)	0.018	3.36 (1.37–8.57)
Anaphylaxis	2 (3.12)	10 (1.29)	0.233	2.45 (0.52–10.91)
Asthma	4 (6.25)	28 (3.63)	0.299	1.76 (0.64–4.81)
Atopic dermatitis	3 (4.68)	28 (3.63)	0.725	1.3 (0.40–4.15)
Conjunctivitis	1 (1.56)	10 (1.29)	0.586	1.2 (0.10–7.16)
Allergic rhinitis	3 (4.68)	34 (4.41)	0.757	1.06 (0.33–3.28)
Insect sting allergy	1 (1.56)	12 (1.55)	>0.999	1 (0.09–5.58)
Drug allergy	3 (4.68)	45 (5.84)	>0.999	0.79 (0.25–2.36)
Animal allergy	0	19 (2.46)	0.388	–
Sibling History				
Conjunctivitis	5 (7.81)	15 (1.94)	0.014	4.26 (1.65–11.20)
Food allergy	9 (14.06)	33 (4.28)	0.003	3.65 (1.63–7.84)
Atopic dermatitis	7 (10.93)	26 (3.37)	0.009	3.51(1.37–8.15)
Drug allergy	10 (15.62)	48 (6.23)	0.009	2.78 (1.36–5.73)
Anaphylaxis	2 (3.12)	9 (1.16)	0.204	2.72 (0.57–10.70)
Allergic rhinitis	10 (15.62)	54 (7.01)	0.023	2.45 (1.20–4.97)
Insect sting allergy	4 (6.25)	23 (2.98)	0.145	2.16 (0.78–6.14)
Animal allergy	6 (9.37)	37 (4.8)	0.132	2.04 (0.87–5.07)
Asthma	4 (6.25)	47 (6.10)	>0.999	1.02 (0.38–2.80)

Each row represents a separate analysis comparing the frequency of the specified risk factor between participants with and without “immediate-type FA, ever”. Acronymous: FA: Food allergy.

## Data Availability

The original contributions presented in this study are included in the article. Further inquiries can be directed to the corresponding authors.

## Supplementary Materials

The following supporting information can be downloaded at: https://www.mdpi.com/article/10.3390/epidemiologia6040085/s1. Figure S1. Public locations visited for sample collection; File S1. Questionnaire Spanish version; File S2. Questionnaire English version.

## Abbreviations

The following abbreviations are used in this manuscript:

CI	Confidence intervals
FA	Food allergy
OR	Odds ratio
