# Supplementary material for: Adult Food Allergy Is an Under-Recognized Health Problem in Northwestern Mexico: A Population-Based Cross-Sectional Survey
Source: Epidemiologia (Basel). 2025 Dec 2;6(4):85. doi: 10.3390/epidemiologia6040085 (PMC12731838; doi:10.3390/epidemiologia6040085)
Supplement: Supplementary file 1 [file epidemiologia-06-00085-s001.zip › epidemiologia-3909993-supplementary.pdf]

Figure S1. Public locations visited for sample collection.

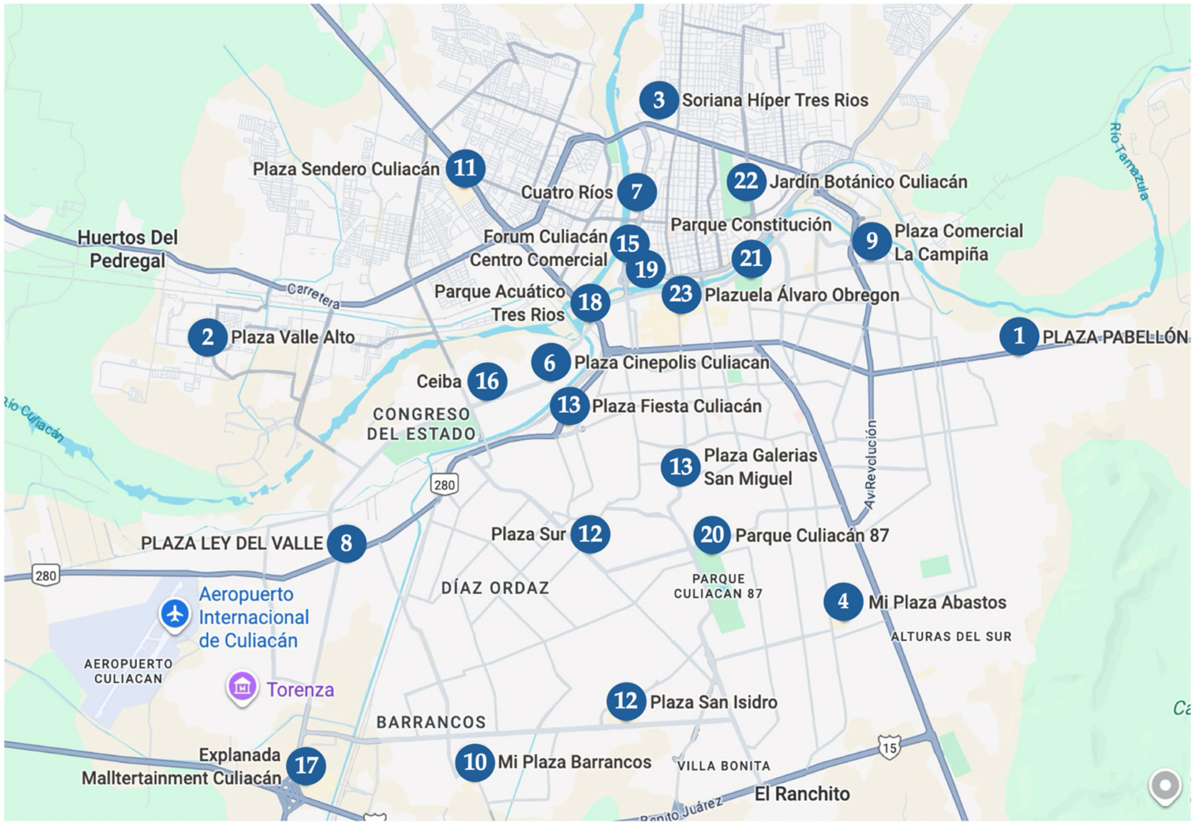

	**PLACES**
**1**	Plaza Pabellon Culiacán
**2**	Plaza Valle Alto
**3**	Soriana Híper Tres Rios
**4**	Mi Plaza Abastos
**5**	Plaza Sur
**6**	Plaza Cinepolis Culiacan
**7**	Cuatro Ríos
**8**	Cinépolis Plaza Azahares
**9**	Plaza Comercial La Campiña
**10**	Mi Plaza Barrancos
**11**	Plaza Sendero Culiacán
**12**	Plaza San Isidro
**13**	Plaza Fiesta Culiacán
**14**	Plaza Galerias San Miguel
**15**	Forum Culiacán Centro Comercial
**16**	Ceiba
**17**	Explanada Malltertainment Culiacán
**18**	Parque Acuatico 3 ríos
**19**	Parque Las Riberas
**20**	Parque Culiacán 87
**21**	Parque Constitución
**22**	Jardín Botánico Culiacán
**23**	Plazuela Álvaro Obregon

Supplementary File S1. Questionnaire Spanish version.

Study: Adult food allergy is a potential emerging health problem in northwestern Mexico: A population-based cross-sectional survey.

Nombre completo: ____ Edad: ___________años

Fecha de nacimiento: ____/_______/________ Sexo Femenino ☐ Masculino ☐ Escolaridad: _________________

Por favor señale con una X la respuesta para cada una de las siguientes preguntas

1. **¿Ha presentado alguna vez una reacción alérgica a algún alimento?**

Marca solo una opción

☐ Si ☐ No

2. **¿Alguna vez un doctor le ha dicho que presenta alguna de las siguientes reacciones?**

Marca solo una opción por fila

a) Asma bronquial	☐ Si	☐ No	☐ No sabe
b) Urticaria	☐ Si	☐ No	☐ No sabe
c) Alergia a la primavera (Rinitis alérgica)	☐ Si	☐ No	☐ No sabe
d) Anafilaxis	☐ Si	☐ No	☐ No sabe
e) Dermatitis atópica	☐ Si	☐ No	☐ No sabe
f) Alergia a insectos	☐ Si	☐ No	☐ No sabe
g) Alergia a los animales	☐ Si	☐ No	☐ No sabe
h) Conjuntivitis alérgica	☐ Si	☐ No	☐ No sabe
i) Alergia a algún medicamento	☐ Si	☐ No	☐ No sabe
j) Otra (especifique)_______________________			

3. **¿Alguna vez usted ha presentado de forma repetida alguna molestia, síntoma o reacción adversa al consumir algún alimento?**

Marca solo una opción

☐ Si **(pase a la siguiente pregunta y continúe)** ☐ No **(pase a la pregunta 21)**

4. **¿Qué alimento(s) le produce(n) molestias o síntomas de forma repetida? (puede contestar más de uno si corresponde)**

Puedes marcar una o más de las opciones

☐ Leche	☐ Cacahuate	☐ Nueces	☐ Pescado	☐ Camarón
☐ Otros mariscos	☐ Soya	☐ Trigo	☐ Huevo	☐ Otro ¿Cuál?

5. **¿Cuál(es) de los siguientes síntomas o molestias presenta de forma repetida cuando consume el (los) alimento(s) mencionado(s) en la pregunta anterior?**

Marca solo una opción

a) Ronchas o sarpullidos que pican ☐ Si ☐ No ☐ No sabe

Marca solo una opción

b) Hinchazón ☐ Si ☐ No ☐ No sabe

¿Dónde?

Puedes marcar una o más de las opciones

☐ Párpados ☐ Labios ☐ Lengua ☐ Cara ☐ Otro:_______________ ☐ No sabe

Marca solo una opción por fila

c) Vómitos	☐ Si	☐ No	☐ No sabe
d) Congestión nasal	☐ Si	☐ No	☐ No sabe
e) Sensación de garganta apretada	☐ Si	☐ No	☐ No sabe
f) Ojos rojos, picazón ocular, lagrimeo	☐ Si	☐ No	☐ No sabe
g) Enrojecimiento de la piel	☐ Si	☐ No	☐ No sabe

¿Dónde?

Puedes marcar una o más de las opciones

☐ Brazos/manos ☐ Cuello/cara ☐ Pecho ☐ Todo el cuerpo ☐ Otro ☐ No sabe

Marca solo una opción por fila

h) Dolor de estómago (abdominal)	☐ Si	☐ No	☐ No sabe
i) Rinitis (secreción nasal, lagrimeo ocular y congestión nasal)	☐ Si	☐ No	☐ No sabe
j) Tos	☐ Si	☐ No	☐ No sabe
k) Dificultad para respirar	☐ Si	☐ No	☐ No sabe
l) Una baja presión que provoca mareo	☐ Si	☐ No	☐ No sabe
m) Desmayo	☐ Si	☐ No	☐ No sabe
n) Diarrea	☐ Si	☐ No	☐ No sabe
o) Picazón de garganta	☐ Si	☐ No	☐ No sabe
p) Silbidos en el pecho	☐ Si	☐ No	☐ No sabe

q) Otros síntomas ¿Cuáles?: ____

6. **¿Cuánto tiempo demoran en aparecer estas molestias o síntomas?** __________________

Si no sabe cuánto tiempo demoran en aparecer estas molestias o síntomas, **"ES MUY IMPORTANTE QUE TRATE DE DARNOS UN TIEMPO APROXIMADO"**:

Marca solo una opción

☐ Menos de 10 min ☐ 10 min-1 hr ☐ 1-2 hr ☐ 2-4 hr ☐ Más de 4 hr ☐ No sabe

7. **¿En cuántas ocasiones ha presentado estos síntomas o molestias?**

Si no recuerda, es muy importante que de un número aproximado:

Número de ocasiones ________________ ☐ *No sabe*

8. ¿En qué lugar(es) han ocurrido de manera accidental la aparición de síntomas o molestias mencionados?

Puedes marcar una o más de las opciones

☐ En casa ☐ En un restaurante ☐ En casa de un familiar ☐ No lo recuerdo ☐ Otro _______________

9. ¿Consultó usted un servicio de urgencia o médico por los síntomas que presentó?

Marca solo una opción

☐ Si ☐ No ☐ No sabe

10. ¿Debió tomar alguno de los siguientes medicamentos debido a los síntomas que presentó?

Puedes marcar una o más de las opciones

☐ Antihistamínicos ☐ Adrenalina ☐ Corticoides ☐ Inhaladores ☐ Suero

☐ No ☐ No sabe ☐ Otro __________________

11. ¿Alguna vez se le indicó como parte del tratamiento la adrenalina inyectable (Adreject o EpiPen)?

Marca solo una opción

☐ Si (Pase a la siguiente pregunta) ☐ No / no sabe (pase a la pregunta 18)

12. Si la respuesta anterior fue Si, ¿Tiene o tuvo un autoinyector de adrenalina?

Marca solo una opción

☐ Si ☐ No ☐ No sabe

13. Si tiene o tuvo un autoinyector de adrenalina, ¿Lo lleva o llevaba el niño(a) siempre consigo?

Marca solo una opción

☐ *Si* ☐ *No* ☐ *No sabe*

14. Si le indicaron un autoinyector de adrenalina y actualmente no lo tiene, ¿Por qué no lo tiene?

Puedes marcar más de una opción

☐ Cree que no es necesario ☐ Basta con evitar el alimento ☐ No se ha vuelto a indicar

☐ El precio es alto ☐ No lo ha encontrado

15. ¿Qué edad tenía cuando por primera vez consumió el(los) alimento(s) que le ocasionan las molestias o síntomas mencionados anteriormente? ___________

Si no recuerda la edad exacta, denos un aproximado por favor

Marca solo una opción

☐ Menos de 1 año ☐ 1-5 años ☐ 6-10 años ☐ 11-15 años ☐ otra _________________

16. ¿Qué edad tenía cuando experimentó por primera vez las molestias? ___________

Si no recuerda la edad exacta, denos un aproximado por favor

Marca solo una opción

☐ Menos de 1 año ☐ 1-5 años ☐ 6-10 años ☐ 11-15 años ☐ otra

17. ¿Actualmente puede consumir los alimentos que le ocasionaron las molestias mencionadas anteriormente sin presentar ninguna reacción?

Marca solo una opción

☐ Si ☐ No ☐ No sabe

18. ¿Alguna vez un profesional de la salud le dijo que las molestias que presentaba se debían a una alergia alimentaria? (Diagnóstico de alergia alimentaria)

Marca solo una opción

☐ Si ☐ No me lo han dicho ☐ No recuerdo si me lo han dicho

19. ¿Qué profesional de la salud le realizó el diagnóstico de alergia alimentaria?

Puede marcar más de una opción

☐ Médico general ☐ Gastroenterólogo ☐ Médico homeópata ☐ Nutriólogo
☐ Alergólogo ☐ Internista ☐ Otro _______________

20. ¿De qué forma le realizaron el diagnóstico?

Puede seleccionar más de una opción

☐ Solo por los síntomas o malestares que presentó
☐ Le pidieron que consumiera el alimento sospechoso para evaluar la aparición de los síntomas (reto oral)
☐ Le hicieron análisis de sangre en laboratorio (anticuerpos)
☐ Le hicieron pruebas en la piel para observar si presentaba enrojecimiento (punción cutánea)

21. ¿Su padre presenta alguna de las siguientes?

Marca solo una opción por fila

a) Asma bronquial	☐ Si	☐ No	☐ No sabe
b) Alergia alimentaria	☐ Si	☐ No	☐ No sabe
c) Alergia a la primavera (Rinitis alérgica)	☐ Si	☐ No	☐ No sabe
d) Anafilaxis	☐ Si	☐ No	☐ No sabe
e) Dermatitis atópica	☐ Si	☐ No	☐ No sabe
f) Alergia a insectos	☐ Si	☐ No	☐ No sabe
g) Alergia a los animales	☐ Si	☐ No	☐ No sabe
h) Conjuntivitis alérgica	☐ Si	☐ No	☐ No sabe
i) Urticaria	☐ Si	☐ No	☐ No sabe
j) Alergia a algún medicamento	☐ Si	☐ No	☐ No sabe

22. ¿Su madre presenta alguna de las siguientes?

Marca solo una opción por fila

a) Asma bronquial	☐ Si	☐ No	☐ No sabe
b) Alergia alimentaria	☐ Si	☐ No	☐ No sabe
c) Alergia a la primavera (Rinitis alérgica)	☐ Si	☐ No	☐ No sabe
d) Anafilaxis	☐ Si	☐ No	☐ No sabe
e) Dermatitis atópica	☐ Si	☐ No	☐ No sabe
f) Alergia a insectos	☐ Si	☐ No	☐ No sabe
g) Alergia a los animales	☐ Si	☐ No	☐ No sabe
h) Conjuntivitis alérgica	☐ Si	☐ No	☐ No sabe
i) Urticaria	☐ Si	☐ No	☐ No sabe
j) Alergia a algún medicamento	☐ Si	☐ No	☐ No sabe

23. ¿Algún hermano o hermana presenta alguna de las siguientes?

Marca solo una opción por fila

a) Asma bronquial	☐ Si	☐ No	☐ No sabe

b) Alergia alimentaria	☐ Si	☐ No	☐ No sabe
c) Alergia a la primavera (Rinitis alérgica)	☐ Si	☐ No	☐ No sabe
d) Anafilaxis	☐ Si	☐ No	☐ No sabe
e) Dermatitis atópica	☐ Si	☐ No	☐ No sabe
f) Alergia a insectos	☐ Si	☐ No	☐ No sabe
g) Alergia a los animales	☐ Si	☐ No	☐ No sabe
h) Conjuntivitis alérgica	☐ Si	☐ No	☐ No sabe
i) Urticaria	☐ Si	☐ No	☐ No sabe
j) Alergia a algún medicamento	☐ Si	☐ No	☐ No sabe

Muchas gracias por participar

Supplementary File S2. Questionnaire English version.

Study: Adult food allergy is a potential emerging health problem in northwestern Mexico: A population-based cross-sectional survey.

Note: The instrument was validated in the Spanish version.

Complete Name: ____ Age: _____________years

Birth date: ______/_______/________ Sex: Female ☐ Male ☐ Education: ________________

Please mark with an X the answer to each of the following questions

1. Have you ever had an allergic reaction to food?

Select only one option

☐ Yes ☐ No

2. Have you ever been told by a doctor that you have any of the following reactions?

Select only one option for each row

a) Asthma	☐ Yes	☐ No	☐ I don't know
b) Hives	☐ Yes	☐ No	☐ I don't know
c) Allergy to the spring (Allergic rhinitis)	☐ Yes	☐ No	☐ I don't know
d) Anaphylaxis	☐ Yes	☐ No	☐ I don't know
e) Atópic Dermatitis	☐ Yes	☐ No	☐ I don't know
f) Insect Alergy	☐ Yes	☐ No	☐ I don't know
g) Animal allergy	☐ Yes	☐ No	☐ I don't know
h) Allergic Conjunctivitis	☐ Yes	☐ No	☐ I don't know
i) Drug allergy	☐ Yes	☐ No	☐ I don't know
j) Other (specify)__________________________________			

3. Have you ever repeatedly experienced any discomfort, symptom, or adverse reaction when consuming food?

Select only one option

☐ Yes **(Go to the next question and continue)** ☐ No **(Go to the question 21 page 5)**

4. **What food causes you repeated discomfort or symptoms? (more than one if applicable).**

Can select one or more options

☐ Milk	☐ Peanut	☐ Nuts	☐ Fish	☐ Shrimp
☐ Others Seafood	☐ Soy	☐ Wheat	☐ Egg	☐ Other ¿which one?

5. Which of the following symptoms or discomfort do you repeatedly experience when consuming the food(s) mentioned in the previous question(s)?

Select only one option

a) Itchy rashes or hives ☐ Yes ☐ No ☐ I don't know

Select only one option

b) Swelling ☐ Yes ☐ No ☐ I don't know

¿Where?

Can select one or more options

☐ Eyes ☐ Lips ☐ Tongue ☐ Face ☐ Other:_______________ ☐ I don't know

Select only one option for each row

c) Vomit	☐ Yes	☐ No	☐ I don't know
d) Nasal congestion	☐ Yes	☐ No	☐ I don't know
e) Throat tightness	☐ Yes	☐ No	☐ I don't know
f) Red eyes, itchy eyes, tearing	☐ Yes	☐ No	☐ I don't know
g) Skin redness	☐ Yes	☐ No	☐ I don't know

¿Where?

Can select one or more options

☐ Arms/hands ☐ Neck/face ☐ Chest ☐ Whole body ☐ Other ☐ I don't know

Select only one option for each row

h) Abdominal pain	☐ Yes	☐ No	☐ Don't know
i) Rhinitis (runny nose, tearing, nasal congestion)	☐ Yes	☐ No	☐ Don't know
j) Cough	☐ Yes	☐ No	☐ Don't know
k) Trouble breathing	☐ Yes	☐ No	☐ Don't know
l) Low blood pressure	☐ Yes	☐ No	☐ Don't know
m) Fainting	☐ Yes	☐ No	☐ Don't know
n) Diarrhea	☐ Yes	☐ No	☐ Don't know
o) Itchy throat	☐ Yes	☐ No	☐ Don't know
p) Wheezing	☐ Yes	☐ No	☐ Don't know

q) Others symptoms ¿Which?: ____

6. How long does it take for these discomfort or symptoms to appear? ___________________

If you do not know how long it takes for these discomforts or symptoms to appear, "**IT IS VERY IMPORTANT THAT YOU TRY TO GIVE US AN APPROXIMATE TIME**"

Select only one option

☐ Less than 10 min ☐ 10 min-1 hr ☐ 1-2 hr ☐ 2-4 hr ☐ More than 4 hr ☐ Don't know

7. How many times have you experienced these symptoms or discomfort?

If you do not remember it, it is very important that you give us an approximate number.

Number of times ________________ ☐ *Don't know*

8. In which places have the previously mentioned symptoms or discomfort occurred accidentally?

Can select one or more options

☐ At Home ☐ In a restaurant ☐ At a relative's house ☐ I don't remember ☐ Other _______________

9. Did you visit an emergency service or see a doctor for the symptoms you presented?

Select only one option

☐ Yes ☐ No ☐ I don't know

10. Did you have to take any of the following medications because of your symptoms?

Can select one or more options

☐ Antihistamines ☐ Adrenaline ☐ Corticoids ☐ Inhalers ☐ Fluids

☐ No ☐ I don't know ☐ Other (which one) __________________

11. Were you ever prescribed injectable adrenaline (Adreject or EpiPen) as part of your treatment?

Select only one option

☐ **Yes (**Go to the next question and continue) ☐ **No /Don't know (**Go to the question **18** page **4)**

12. If the previous answer was yes, do you have or have you had an adrenaline autoinjector?

Select only one option

☐ Yes ☐ No ☐ Don't know

13. If you have or had an adrenaline autoinjector, do you carry it with you?

Select only one option

☐ Yes ☐ No ☐ Don't know

14. If you were given an autoinjector of adrenaline and currently do not have it, why do you not have it?

Can Select one or more options

☐ I believe there is no need ☐ I just avoid the food ☐ It has not been re-indicated

☐ The price is high ☐ I have not found it

15. How old were you when you first ate the food(s) causing the discomfort or symptoms mentioned above?

If you do not remember the exact age, please give us an approximate.

Select only one option

☐ Less than 1 year ☐ 1-5 years ☐ 6-10 years ☐ 11-15 years ☐ other _________________

16. How old were you when you experienced the discomforts for the first time?

If you do not remember the exact age, please give us an approximate

Select only one option

☐ Less than 1 year ☐ 1-5 years ☐ 6-10 years ☐ 11-15 years ☐ other__________________

17. Can you currently consume the foods that cause you the discomfort mentioned above?

Select only one option

☐ Yes ☐ No ☐ Don't know

18. Has a health professional ever told you that your discomfort was due to a food allergy? (diagnosis of food allergy).

Select only one option

☐ Yes ☐ They didn't say it ☐ Don't remember if I was told

19. Which health professional diagnosed you with a food allergy?

Can Select one or more options

☐ General practitioner ☐ Gastroenterologist ☐ Homeopath ☐ Nutriologist

☐ Allergist ☐ Internist ☐ Other _______________

20. How was the diagnosis made?

Can Select one or more options

☐ Just because of the symptoms or discomfort you presented

☐ Asked to eat the suspect food to assess the onset of symptoms (oral challenge)

☐ Laboratory blood tests (antibody)

☐ Skin tests to see if you had redness (prick test)

21. Does your father have any of the following?

Select only one option for each row

a) Asthma	☐ Yes	☐ No	☐ Don't know
b) Food allergy	☐ Yes	☐ No	☐ Don't know
c) Allergy to the spring (allergic rhinitis)	☐ Yes	☐ No	☐ Don't know
d) Anaphylaxis	☐ Yes	☐ No	☐ Don't know
e) Atópic Dermatitis	☐ Yes	☐ No	☐ Don't know
f) Insect Alergy	☐ Yes	☐ No	☐ Don't know
g) Animal allergy	☐ Yes	☐ No	☐ Don't know
h) Allergic Conjunctivitis	☐ Yes	☐ No	☐ Don't know
i) Hives	☐ Yes	☐ No	☐ Don't know
j) Drug allergy	☐ Yes	☐ No	☐ Don't know

22. Does your mother have any of the following?

Select only one option for each row

a) Asthma	☐ Yes	☐ No	☐ Don't know
b) Food allergy	☐ Yes	☐ No	☐ Don't know
c) Allergy to the spring (allergic rhinitis)	☐ Yes	☐ No	☐ Don't know
d) Anaphylaxis	☐ Yes	☐ No	☐ Don't know
e) Atópic Dermatitis	☐ Yes	☐ No	☐ Don't know
f) Insect Alergy	☐ Yes	☐ No	☐ Don't know
g) Animal allergy	☐ Yes	☐ No	☐ Don't know
h) Allergic Conjunctivitis	☐ Yes	☐ No	☐ Don't know
i) Hives	☐ Yes	☐ No	☐ Don't know
j) Drug allergy	☐ Yes	☐ No	☐ Don't know

23. Any brother or sister have any of the following?

Select only one option for each row

a) Asthma	☐ Yes	☐ No	☐ Don't know
b) Food allergy	☐ Yes	☐ No	☐ Don't know
c) Allergy to the spring (allergic rhinitis)	☐ Yes	☐ No	☐ Don't know
d) Anaphylaxis	☐ Yes	☐ No	☐ Don't know

e) Atópic Dermatitis	☐ Yes	☐ No	☐ Don’t know
f) Insect Alergy	☐ Yes	☐ No	☐ Don’t know
g) Animal allergy	☐ Yes	☐ No	☐ Don’t know
h) Allergic Conjunctivitis	☐ Yes	☐ No	☐ Don’t know
i) Hives	☐ Yes	☐ No	☐ Don’t know
j) Drug allergy	☐ Yes	☐ No	☐ Don’t know

Thank you for your participation
